# Education for children and adolescents living with disabilities in sub–Saharan Africa—The gaps and opportunities

**DOI:** 10.3389/fpubh.2022.979351

**Published:** 2022-09-07

**Authors:** Pauline Samia, Katherine Oyieke, Barnabas Kigen, Susan Wamithi

**Affiliations:** ^1^Department of Paediatrics and Child Health, Medical College, Aga Khan University, Nairobi, Kenya; ^2^Brain and Mind Institute, Aga Khan University, Nairobi, Kenya; ^3^Department of Public Health and Primary Care, Ghent University, Ghent, Belgium; ^4^Department of Child Health and Paediatrics Medical College, Moi University, Eldoret, Kenya

**Keywords:** early childhood education, school readiness, adolescent education, disability, Africa

## Introduction

The World Health Organization (WHO) defines disability as an umbrella term that covers impairments, activity limitations, and restrictions in participation (1). Disability is not considered a health problem, but rather an interaction between a person's body functions and features of the environments in which they live (1). WHO report a higher prevalence of severe and moderate disabilities in Africa compared to other regions (1). The United Nations Children's Fund (2021) provides a global estimate of 230 million children, ages 0–17 years, living with a disability with 28.9 million children found in Eastern and Southern Africa (2). More than half of these children live in rural settings and only about one third attend a primary school (1). Given the high birth rate of 22.6 births per 1,000 people in East Africa, and successful implementation of interventions that have significantly reduced the under-5 mortality rate in this region, the prevalence of childhood disability can only increase over time (3, 4). This is a pertinent current and future issue given that the estimated likelihood of a child having a disability before their fifth birthday is 10 times higher than the likelihood of dying (377.2 vs. 38.2 per 1,000 live births) (5).

The UN Sustainable Development Goals (6), place early childhood development as an international priority. Specifically, target 4.2 sets out a clear mandate to “ensure that all girls and boys have access to good-quality early childhood development” with specific global indicators measuring the proportion of children under 5 years of age, who are developmentally on track in health, learning and psychosocial wellbeing (7). To achieve optimum early childhood development, the Sustainable Development Goals (SDGs) require regular monitoring of all children's health and wellbeing (7, 8).

Successful models of inclusive education have been implemented in low and middle income countries (LMIC) such as Malaysia which has systematically provided for training of special education teachers from 1990 and created a department for special education in 1995. This was followed a chapter on special education in the education act in 1996 and the education rules that established special schools as well as integrated and inclusive education programs (9). Malaysia implemented the “zero reject” policy in 2019 which aims to ensure that children living with disability can be enrolled in any government or government assisted school of their choice (10).

**Figure 1:**
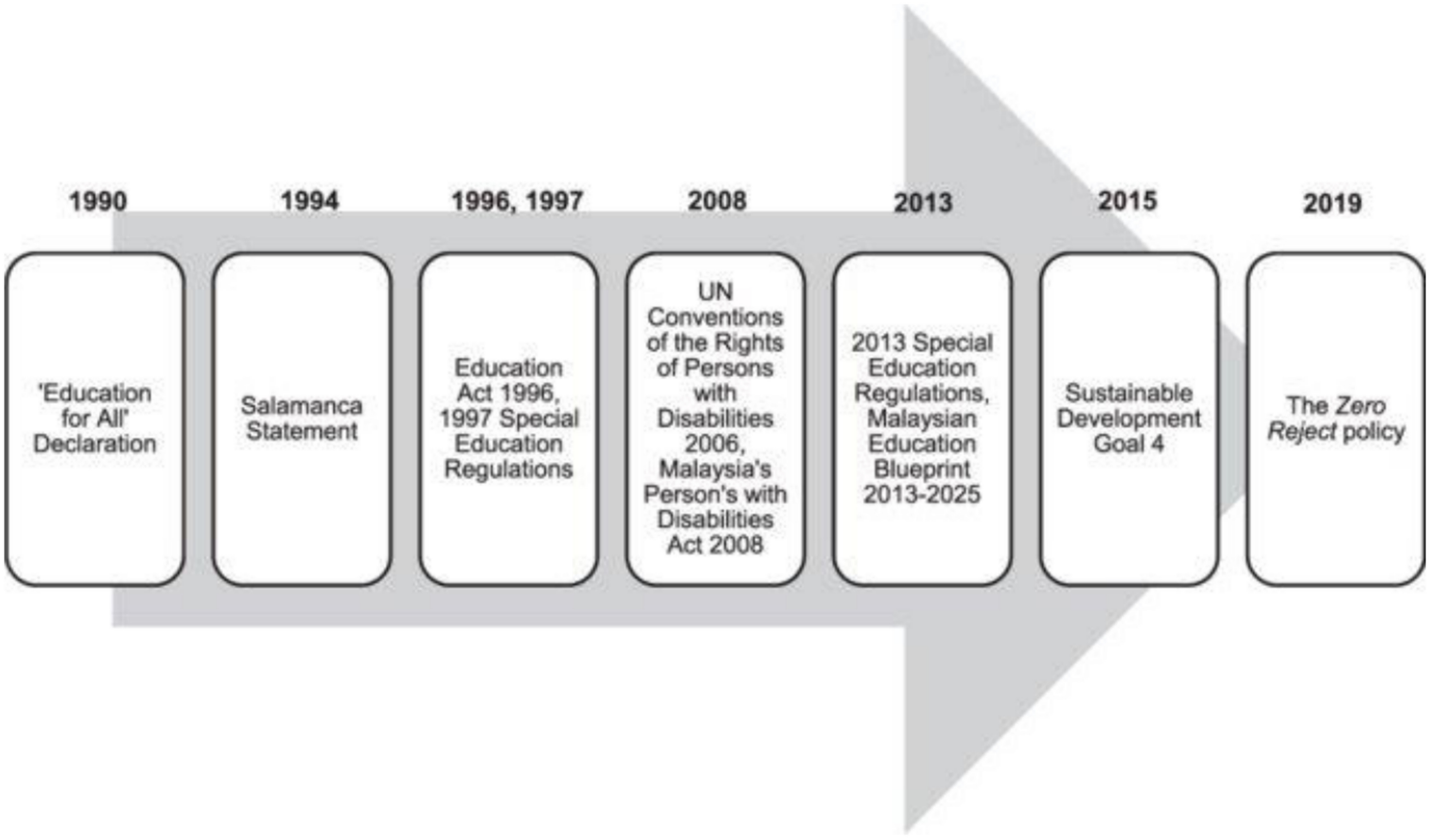
The evolution of inclusive education in Malaysia. Ref. Chin (10).

This exemplary evolution in inclusive education in Malaysia is summarized in Figure 1 below.

In one global data set on children with developmental disorders in Africa, the most common disabilities reported were hearing and visual impairments, intellectual disability and autism spectrum disorder (4). Illiteracy among adults living with disability in Africa compromises potential personal independence, desired social interactions, and exposes this group to exploitation (11).

To adequately meet the needs of CALWD Sub-Saharan Africa needs to refocus its efforts. This redefined focus requires integrated interventions including measures to reduce occurrence of developmental disabilities by targeting preventable biological and environmental contributors, such as sub-optimal perinatal care and economic deprivation; promotion of early diagnosis of disabilities coupled with timely interventions delivered during the time sensitive periods of early brain development; and finally support for wide-ranging, accessible and impactful interventions one of which is inclusive education (3, 11–14). Inclusive education also demands provision of assistive technologies inclusive of hardware and software, and an accommodating environment that allows the best possible attainment for these CALWD (8, 15, 16). This calls for a progressive policy framework driven by governments and relevant partners for realization of these demands (11). Special attention is required for the girl-child living with disability. In East Africa she is much less likely to remain and complete her education compared to her male counterparts and especially so if she hails from an ultra-poor background (10). In addition, cultural norms, biological factors, insecurity, climate change and unprecedented events such as the COVID-19 pandemic have all further contributed to this occurrence (12).

## The current state of schools in Africa in accommodating CALWD

Education in young children provides an opportunity to refine developmental abilities that contribute toward highest attainable level of personal independence (8). Development of language, cognition, motor abilities and quality of social interaction progresses rapidly throughout the early years of life. It is during this time that tailored education efforts are most likely to be most impactful for all children. Unmet developmental potential in children and young people has social and economic implications for individuals, families and the community at large by negating potential contributions and independence (8, 14, 17).

Children and adolescents who live with physical disability require specific physical accommodations to allow them participate in all-inclusive education settings. Where possible co-location of therapy supports within the school allows CALWD access these services with minimal compromise to school attendance. Such considerations are rarely ever applied within the majority of schools in sub–Saharan Africa and South Asia (8, 12, 15).

A study from South Africa observed that a facility for deaf-blind learners was available but educators and their assistants were ill-equipped to meet the diverse learning needs of these students and had had minimal access to skills upgrade systems which negatively impacted their capacity for optimal skills transfer to learners (18–20). Poor availability of speech language pathologists particularly in East Africa negatively impacts on the possibility for hearing impaired children to receive interventions that prepare them for formal education (21).

In Africa an estimated 350,674 children below 15 years of age are blind and many more are living with undiagnosed low vision (22, 23). Children and adolescents with visual impairment require specifically trained teachers, equipment orientation interventions and ophthalmology services that provide enhancements to make reading possible. The evidence base demonstrates that these support are largely unavailable contrary to the convention on the rights of persons with disability which envisages inclusive education leading to opportunity loss for education for such children who are otherwise capable of learning (24). Gender specific exclusions have also been observed in Africa with school enrolment of visually impaired girls being lower than that of boys (24). Overall transition rates from primary to secondary school for visually impaired children and adolescents is also low in Africa (20).

Instances of bullying and intentional physical violence toward vulnerable children and adolescents with various disabilities have been reported (25, 26). Physical violence from school staff is a particularly common experience among children under 18 years in schools in Kenya and Tanzania (25). Indeed, the frequency of violence toward CALWD is higher than that reported by typically developing children (25, 26). School based interventions such as the “Good-school toolkit” have been effectively utilized to reduce violence toward adolescents living with disability (27, 28).

## School readiness and optimizing education for CALWD in Africa

Africa has a predominantly young population and has opportunity to improve economic outputs and quality of life for its communities by empowering CALWD through provision of relevant and contextually appropriate education (29). According to the National Educational Goals Panel, a child's school readiness is dependent on supportive families, communities and schools. Children's school readiness consists of five components; physical health and motor development, social and emotional development, language development, approaches to learning, cognition, and general knowledge (30). Health care providers are in close contact with families prenatally up to young adulthood providing opportunities to optimize school readiness by supporting these five components from the very beginning (30). With the exception of Southern Africa, minimal data exists on efforts to ensure school readiness for CALWD (31, 32).

Early diagnosis of childhood onset disability is a critical first step in improving health related and other outcomes for this population. Studies from south Asia and Africa have demonstrated that assessments lacking adaptation to specific cultural contexts can lead to inaccurate interpretation of performance (33–35). Utilization of locally developed and validated assessment tools as well as inclusion of parents in assessment of CALWD would help identify and place CALWD in appropriate educational settings (33–35). Parents may act as teachers, partners, decision makers and advocates for CALWD and should be continually involved even when their own literacy skills are low (36, 37).

Collaborative models involving parental inputs, training of special education teachers and providing inclusive education that also co-locates therapists operating in the same setting would bring African countries closer to achieving effective education for CALWD (38). To achieve these wide-ranging measures, interventions including policy development and implementation as and changes in social-cultural attitudes toward education for CALDW would be required (9, 10, 27). African countries would need to commit advancements to improve the understanding of the general public regarding education for CALWD in order to realize the vision of an inclusive education (17, 27, 38).

Peer support and social interactions between individuals with disabilities and typically developing children have been shown to have significant positive impacts on the lives of children with disabilities (39). Typically developing children and adolescents better understand the unique needs and strengths CALWD have and can better advocate and accommodate them in their current and future operations when both groups participate in an inclusive education setting (14). This leads to a more cohesive society where CALWD and their families are “seen,” “heard” and have sense of belonging. Africa has the opportunity to educate its communities better on the needs and benefits of inclusive education (27, 38). These understandings would reduce stigma, emotional and physical abuse and eventually improve advocacy for individuals and institutions that support CALWD.

Ratification of Convention on the Rights of Persons with Disabilities by the remaining African states will form a basis for ensuring support for these vulnerable persons (2, 11, 38, 40).

## Conclusion

Childhood disability in Africa is currently a significant concern with the numbers of those affected expected to increase over time. This calls for a redefined attention to integrated and multilayered approaches to reduce occurrence and impact of developmental disabilities. Current school environments in Africa largely do not cater to the social, physical, and technological accessibility to education that fosters long term inclusivity of CALWD. In the final analysis this this negates possibility for future independence and positive contribution to society for CALWD. Parents and healthcare workers should be facilitated to participate in nurturing care, assessment and identification of young children with disability which turn fosters school readiness increasing the possibility for CALWD to participate in education. Utilization of contextually appropriate and validated tools to identify CALWD in Africa will contribute to education related advocacy efforts and encourage policy makers fully implement the goals envisioned in SDG 4.

## Author contributions

PS and SW provided the concept for the opinion piece. All authors contributed to the submission and approved the final version.

## Conflict of interest

The authors declare that the research was conducted in the absence of any commercial or financial relationships that could be construed as a potential conflict of interest.

## Publisher's note

All claims expressed in this article are solely those of the authors and do not necessarily represent those of their affiliated organizations, or those of the publisher, the editors and the reviewers. Any product that may be evaluated in this article, or claim that may be made by its manufacturer, is not guaranteed or endorsed by the publisher.
